# Education Research: Qualitative Assessment of Virtual Teaching of the Neurological Examination to Students Reveals Importance of Technique, Process, and Documentation

**DOI:** 10.1212/NE9.0000000000200083

**Published:** 2023-07-14

**Authors:** Sandra Reiter-Campeau, Stuart Lubarsky, Colin H. Chalk, Asli Buyukkurt, Myriam Levesque-Roy, Ana Clouatre, Diana Benea, Tasnia Rahman, Fraser Moore

**Affiliations:** From the Department of Neurology and Neurosurgery (S.R.-C., S.L., C.H.C., A.B., M.L.-R., T.R., F.M.), Institute for Health Sciences Education (S.L., C.H.C., F.M.), and Faculty of Medicine (A.C., D.B.), McGill University, Montreal, Quebec, Canada.

## Abstract

**Introduction:**

Virtual teaching sessions during the coronavirus disease 2019 pandemic were challenging for students and teachers but were also an opportunity to find creative ways to teach physical examination skills, including the neurologic examination. We examined expert opinions of the pros and cons that arise using a virtual platform to teach the neurologic examination and strategies to best address these challenges.

**Methods:**

This was a qualitative study incorporating a focus group of faculty and resident neurologists. Data were coded using conventional content analysis. An interpretivist, social constructionist approach was used to look for interesting or novel ideas, rather than testing a specific hypothesis. Three independent auditors performed a dependability and confirmability audit to confirm that the themes accurately reflected the data.

**Results:**

A single focus group was used. Four of the 6 participants were faculty neurologists and 2 were neurology residents. Five themes were identified: (1) learning the neurologic examination is complex, (2) lack of physical contact is the most important drawback of virtual teaching, (3) virtual teaching can effectively emphasize the organization of the examination, (4) virtual sessions can facilitate combined teaching of technique and demonstration of abnormalities, and (5) virtual platforms do not necessarily imply reduced participation.

**Conclusion:**

Teaching the neurologic examination is a multifaceted process that should emphasize not only technique but also an overall approach to performing and documenting the examination. Many aspects of the neurologic examination can be appropriately taught virtually using various strategies, although there may always be some limitations. Virtual education can play a useful role for future curriculum design and global education.

The coronavirus disease 2019 (COVID-19) pandemic forced medical schools to move the bulk of their teaching sessions to a virtual format.^1^ One aspect for which this has been most challenging is teaching the physical examination.^2^ Demonstrating physical examination techniques online or using recorded videos are often poor substitutes for explaining a technique and observing an examination in person. Depending on local protocols, students may not even have had the opportunity to work in pairs to practice examination techniques.

The neurologic examination consists of many different elements.^3,4^ Students often cite the complexity of the neurologic examination. Some elements can be demonstrated and evaluated virtually, such as finger-to-nose testing for limb ataxia. For other elements, techniques are less obvious. For example, when testing muscle tone and reflexes, an instructor may need to help a student position their hands and the limbs of the subject; when testing ocular movements, the speed at which the student moves their target and the depth of the target from the subject may be difficult to judge over an Internet connection.

Publications have concentrated on how practicing physicians can perform virtual examinations,^5,6^ but there is limited evidence to guide remote *teaching* of complex clinical skills including the neurologic examination. Virtual patients have been used to teach history taking,^7,8^ as an alternative to lecture-led small group case-based teaching,^9^ and as a way to demonstrate abnormal neurologic examination findings.^10^ A review of virtual, augmented, and alternate reality methods described their applicability for procedural and communication skills but not for the physical examination.^11^ Virtual platforms have been used to practice history taking and data interpretation skills in preparation for objective structured clinical examinations (OSCEs)^12^ and to deliver OSCEs that included parts of the neurologic examination requiring only inspection.^13^ Recorded videos of examination techniques have been used with a flipped classroom approach to supplement in-person instruction.^14^

At McGill University, medical students are introduced to the neurologic examination during eight 2-hour small group teaching sessions in the fall of their second year. Students practice examination techniques on each other under the supervision of a faculty or resident tutor. In 2020, these small group teaching sessions were held virtually via video conferencing (with each student participating via their individual connection). This change presented a challenge to tutors, but it was also an opportunity to find creative ways to teach the neurologic examination.^15^ The purpose of this study was to use expert consensus to identify how the neurologic examination could be taught in a virtual format.

## Methods

### Study Design

This was initially designed as a mixed methods study. An e-mailed written survey was sent to tutors asking them about the strategies they used to teach the neurologic examination in a virtual format. We intended to follow this with recorded one-on-one Zoom meetings with tutors and use a Delphi approach^16^ to identify the optimal strategy for teaching each element of the neurologic examination. However, from the initial quantitative data collected, it was evident that tutors had not used a large number of *element-specific* strategies (e.g., one strategy for teaching how to examine visual fields, another strategy for ocular movements, another for muscle tone, etc…). We modified our methods. Rather than presenting quantitative data about each individual element, we focused on a qualitative approach to the question of how to best teach the neurologic examination as a whole in a virtual format. Our broader goal was to understand what this could inform us about teaching the neurologic examination in any format.

### Context

As part of the 8-week “Human Behavior” course in the second year of the 4-year medical school curriculum at McGill University, students participate in eight 2-hour small group teaching sessions. In the first hour, they discuss a case (a history and neurologic examination findings are provided). The emphasis is on localization of the neurologic problem. The second hour is used to teach elements of the examination for which technique is important. Specific topics include the following: muscle tone, stretch reflexes, tests of coordination (finger-to-nose, rapid alternating movements, heel-to-shin), different sensory modalities, gait, mental status (cognition), pupils, visual fields, and ocular movements. Students previously received a lecture explaining the examination as a whole and were provided with links to 2 videos of a screening neurologic examination (one showing an uninterrupted examination and a second explaining each individual element).

Each group consists of 12–15 second-year medical students and a resident or faculty tutor. Examination techniques are explained, and students are encouraged to practice techniques on each other while the tutor circulates in the room and provides feedback or answers questions. In 2020, these sessions all occurred virtually. Students connected individually and were not paired up. The format of the sessions was otherwise unchanged. The tutors were encouraged to be creative in teaching the neurologic examination but did not receive more specific instructions or a preparatory session.

### Participants and Quantitative Data Collection

All 15 tutors in 2020 (11 faculty and 4 residents) were invited to respond to an e-mailed survey (eAppendix 1, links.lww.com/NE9/A38) asking them about their approach to teaching the neurologic examination virtually and if they had suggestions for future sessions. Two of the questions (5 and 6) asked about the strategies that were used for individual elements of the examination (e.g., what strategy was used for muscle tone? For reflexes? etc…). The remaining questions asked more generally about virtual teaching strategies. The survey was directly e-mailed to the participants and was not pilot tested. Survey responses were collected by one of the authors (F.M.) and then circulated to the remaining authors for feedback.

We received 9 responses to the survey. Of these, only 3 provided complete answers to questions 5 and 6 (eAppendix 1, links.lww.com/NE9/A38) that asked how the tutor taught each individual element of the examination. A further 3 responses gave incomplete answers while the remaining 3 responses discussed only strategies in general.

### Qualitative Data Collection

Because the survey did not identify a large number of *element-specific* strategies, we modified our methods to focus on broader strategies used to teach the neurologic examination. A focus group of 5 tutors who participated in the course in both 2020 and 2021 was organized. The recruitment and sampling method was convenience sampling because individuals who tutored in both 2020 (when teaching was virtual) and 2021 (when teaching was in-person) were specifically selected. It was hoped that their experience with returning to in-person teaching (in 2021) after virtual teaching (in 2020) allowed them to reflect on and contrast the 2 methods. These 5 tutors are all coauthors on this study (S.L., C.H.C., and M.L.-R. are faculty Neurologists at McGill; A.B. and S.R.-C. are McGill Neurology residents).

The senior author (F.M.) contacted the 5 tutors and provided them with a summary of the survey results. They were asked to reflect on these results as well as on their recent experience returning to in-person teaching of the neurologic examination. The focus group meeting was held virtually in January 2022 and was facilitated by the senior author (F.M.). Participants were aware of the purpose of the study. Free text responses from the initial questionnaire were used to inform the approach to questions explored in the focus group. Five main questions were discussed:

1. What can (realistically) be done virtually?
2. What (absolutely) cannot be done virtually?
3. What suffers the most from virtual sessions?
4. Is there anything that works really well virtually?
5. How would we do this next time? (If we had to return to virtual teaching)

Participants had the opportunity to provide their perspectives. Rather than interviewing each participant in turn, the focus group took the form of a discussion and exchange of ideas. Individuals were encouraged to respond to or expand on the ideas of their colleagues. Comparing and contrasting the experience in 2020 (virtual) and 2021 (in-person) was part of the discussion that occurred, but participants were not explicitly asked to do this (e.g., “what was easier or better this past year compared with 2020?”).

The focus group lasted until participants were satisfied that all themes and comments had been explored. This took 45 minutes. A specific method was not used to assess data saturation.

### Data Analysis

The focus group was recorded and transcribed by the senior author (F.M.). He also took field notes during the meeting. He has been a practicing Neurologist since 2003, is an Associate Professor in the Department of Neurology and Neurosurgery at McGill University, is the faculty leader for the Human Behavior course, and has been a member of the McGill Institute for Health Sciences Education (previously the Center for Medical Education) since 2004.

The senior author reviewed and coded the data using conventional content analysis.^17,18^ Key words or thoughts were highlighted without initially attempting to identify themes. A second review was performed to code key ideas. Possible themes were noted in a separate document using an inductive approach. The methodological orientation was considered to be an interpretivist, social constructionist approach. The emphasis was on looking for interesting or novel ideas and approaches to the problem, not on testing or confirming hypotheses. For this reason, we did not specifically seek out comparisons between virtual and in-person teaching for analysis.

The data were reviewed again to ensure that possible themes accurately reflected the data and that no important ideas had been excluded. We identified 5 major themes. A final review of the data was performed to identify specific quotes that illuminated the themes.

Three independent auditors (A.C., D.B., and T.R.) performed a dependability and confirmability audit^19^ to confirm that no important themes were missing and that the themes accurately reflected the data. Each auditor reviewed the data collected (responses to e-mailed questionnaires and the transcript of the focus group recording) and the draft results. The auditors had not participated as tutors and did not take part in the data analysis. A. Clouatre was a second-year medical student who was a student in the Human Behavior course in 2021 (when small groups were in-person). D. Benea was a third-year medical student who was a student in the Human Behavior course in 2020 (when small groups were virtual). T. Rahman was a fourth-year medical student who was a student in the Human Behavior course in 2019 (when small groups were in-person). The auditors were also encouraged to provide personal comments and perspective based on their experience as students in the course.

After a review of the auditor's comments, the manuscript was edited by the senior author and sent to the 5 tutors who participated in the focus group (S.L., C.H.C., A.B., S.R.-C., and M.L.-R.) along with a transcript of the focus group. They were invited to comment on the draft with reference to their own analysis of the data collected. Based on the comments received, the document was modified and resubmitted to each of the 5 tutors who participated in the focus group. Any areas of disagreement were resolved by consensus among these 5 tutors and the senior author.

### Standard Protocol Approvals, Registrations, and Patient Consents

The study was approved by the Institutional Review Board of the Faculty of Medicine and Health Sciences of McGill University. Informed consent was obtained from all participants.

### Data Availability

Anonymized data will be shared by request from any qualified investigator.

## Results

### Survey Results

Responses to the e-mailed survey were obtained from 9 of the 15 small group tutors who participated in 2020. Reasons for not responding were not sought. The surveys received did not provide detailed responses about strategies used to teach each individual element of the neurologic examination, and thus, quantitative data regarding each element cannot be presented. The survey responses focused on strategies for teaching the neurologic examination as a whole in a virtual format. This led to modification of the planned methods, as described earlier. Table provides a list of different strategies that survey participants used. The table is inclusive and lists any strategy that was used by 1 or more of the 9 tutors who responded to the survey and which they included in their response.

**Table:** Strategies for Teaching the Neurologic Examination in a Virtual Format

1. Arrange in-person (one-on-one) sessions with students
2. Have the tutor examine a volunteer such as a family member
3. Have the tutor examine a live patient
4. Pair groups such that 2 tutors are together and can demonstrate the examination on each other, while their students watch via the same Zoom call.
5. Show videos that demonstrate the neurologic examination
6. Show videos that demonstrate abnormalities on the neurologic examination
7. Quiz students about the examination (steps, order, technique) before showing a video
8. Show videos of poor examination technique (as contrast)
9. Ask students to “examine” the tutor via video conferencing (taking turns giving instructions to the tutor about what tasks to perform)
10. Use props such as dolls or a mannequin
11. Have students watch videos in advance and then use group time to quiz them
12. Teach the examination as a whole during whole class sessions and then use small group time to follow-up and discuss difficult or problem areas.
13. Have students prepare videos of themselves examining each other (or family members) in advance and then show the videos during a small group session and give feedback.

### Thematic Analysis

Five main themes emerged from the focus group and are discussed further. Quotations are identified by participant (P1, P2, P3, P4, or P5).

#### What Does It Mean to Learn the Neurologic Examination?

Through discussion of strategies to teach the neurologic examination virtually, it became evident that learning the neurologic examination is about more than learning a series of elements or actions to perform. Students must learn the following:

1. What elements to do
2. In what order to do them
3. The technique for performing each step
4. When and how each step can be abnormal
5. How to document the examination

The discussion also highlighted the nuances that exist within each of these steps. For example, teaching the neurologic examination often begins with teaching a screening examination but can also cover teaching how to adapt this in specific clinical situations:

Learning particular approaches to certain patients,… for example, in parkinsonism, and what variations of the examination do we do. (P1)

Another issue was the importance of striking a balance between presenting the examination “in small, digestible chunks” while still being aware that:

…we (over)estimate…how well-prepared students are just to go through a systematic approach. (P4)

It can be tempting to concentrate on teaching proper technique or explaining the importance of the individual elements of the examination. However, students also need to be able to visualize how these elements can fit together into a systematic approach at the bedside. Thus, both instruction regarding individual elements and a broader overview that emphasizes an approach are important.

#### Lack of Physical Contact Is the Most Important Drawback of Virtual Teaching

Certain techniques such as the examination of tone or reflexes require touch as well as observation. These are what are most difficult to teach virtually:

Even if (they) see someone do tone on an upper extremity, I feel like it is difficult to teach unless they do it themselves. (P1)

One of the most valuable parts (is) having them practice on each other and you…give live feedback on how they can improve their technique. (P2)

Learning an examination technique also often includes adjusting to being in close contact with and touching another person:

the big barrier is not so much learning the technique because it is just getting used to getting close to the patient and putting hands on a patient. (P5)

In neurology, the positioning of the examiner relative to the patient and the amount of contact required can vary throughout the examination. Learning when to touch a patient, and how to do it with sensitivity, can be intimidating for students. This could be done virtually by asking students to prepare their own videos of examination technique in advance:

You give them an assignment saying you two have to demonstrate tone together and they could…demonstrate it wherever they feel comfortable, prerecord it, and share it…people can give them feedback, and comments, it would be more engaging than showing videos. (P1)

The instructor can help the students feel comfortable approaching an examination subject by giving feedback and making it explicit that many students struggle with learning how to do this. However, asking students to work together to prepare videos (or having 2 instructors work together, as suggested in the survey results) would require greater accommodation regarding sanitary precautions (and scheduling) on the part of students and/or tutors.

#### Virtual Teaching Can Emphasize the Organization of the Examination

Learning what examination elements to include, an order in which they can be done, and how to document each step can all be explained or shown virtually:

Learning particular approaches to certain patients…and what variations of the examination do we do in that case are the things that they would learn the best over Zoom. (P1)

Learning how to document the examination can then be used to emphasize an order to the examination:

…showing them how we document… will enforce that structure. (P1)

Alternatively, all or part of an examination could be shown and students could be asked how they would document it. This could facilitate learning (by doing rather than seeing).

#### Virtual Sessions Can Combine Teaching of Technique and Demonstration of Abnormalities

How each step of the neurologic examination can be abnormal is something that might be more easily shown virtually:

Even during a clinical rotation, they could go through easily…and not see certain things. Even certain common things… (P4)

Videos could ensure that *every student* has the opportunity to see how an individual examination element could be abnormal. There is, however, a danger in covering abnormalities in too much detail. For example, “with extraocular movements, there are so many things that you are looking at” (P2) and thus many possible abnormalities that could be described. Rather than simply having sessions in which those abnormalities are shown, it might be better “if there was a way to integrate some of those slides, or some of those videos” (P3) of abnormalities into an explanation of the technique for that specific element. A single example of an abnormal test could be used to teach how to perform the test, rather than asking novice students to become comfortable with multiple abnormalities.

#### Virtual Platforms Do Not Necessarily Imply Reduced Participation

One of the difficulties with virtual platforms is that:

…unmuting, turning on the camera, it is just like another layer and for students who are very shy and (for whom) it already takes some effort to pull them in, I found that quite challenging. (P2)

However, a virtual platform could potentially enhance student interaction when they are presented with abnormalities or videos of peers performing the examination:

enlist audience participation,…(ask) what do you think of this, what do you think of…that…there’s tons that goes on in the chat. (P4)

A virtual platform has that additional advantage of easily permitting discussion *between students* in the chat function, whereas having students talk among themselves during an in-person session might be noisy and distracting. The chat function can empower students who otherwise feel too reserved to participate.

Other potential strategies include asking students to verbally explain the neurologic examination prior to demonstrating it, critiquing each other's demonstration (or a video) of the neurologic examination, and promoting group studying by sharing common resources:

…if everyone was kind of using the same site (…) the students would be more likely to (…) share, or study together. (P1)

Increased participation and collegiality may have greater value at this stage of training than an overemphasis on learning more technical aspects of the examination such as tone and reflexes, which may require the experience of ongoing practice.

## Discussion

It remains to be seen what role virtual learning will have in medical school as we move beyond the peak of the COVID-19 pandemic. A return to in-person teaching of the neurologic examination has been and will be celebrated by learners and teachers alike. However, there is evidence that students perceive virtual education as beneficial^20^ and virtual reality will likely have an increasing role in medical education.^21^ Virtual learning could be part of ongoing strategies to improve educational accessibility^22^ and eliminate gaps in education.^23^ This could also make it easier to standardize what each individual student is exposed to. The ease of integrating virtual strategies^24^ may ensure that it has an important role going forward as we move out of the COVID-19 pandemic.

In this context, we anticipate that our results will be valuable in a number of ways. The first is that they will provide some guidance for the individual teacher helping learners with the neurologic examination. This may include novel strategies, the importance of teaching not just technique but also an approach to performing and documenting the examination, and acknowledging the difficulty some students may have with physically approaching a patient.

Rather than attempting to be “all-inclusive” and teach everything about the examination in one setting or format (such as virtual small groups), it might make more sense to diversify the teaching. After all, the first theme identified here was that learning the neurologic examination is about more than technique; it includes learning what to do, an order, technique, potential abnormalities, and documentation.

One approach based on our results could be as follows:

1. Present the steps of the neurologic examination to students in different formats. This might include a reference document that lists the steps, a whole class session (or sessions) that demonstrates an entire examination, and/or a reference video that demonstrates the examination.
2. While presenting the steps, emphasize the importance of and rationale for using a consistent order.
3. Provide an example (or examples) of how to document the examination. This could also be used to emphasize the overall structure and order.
4. Have students or pairs of students each prepare videos of examination techniques in advance. Use the virtual small group time to show the videos and have the other group members critique or ask questions about each video. The tutor could have a volunteer or patient with them to demonstrate techniques in real time, as needed. This could permit the instructor to contrast their positioning and placement relative to the patient with what the students showed in their video, while acknowledging the difficulty that students might feel with approaching and touching a patient.
5. Share videos of selected abnormal findings. Students could be encouraged to communicate their thoughts in the chat. Alternatively, these could be presented in whole-class sessions and/or students could be provided with resources to review them in greater detail later.
6. Providing additional resources for students to consult on their own time including tables, pictorials, and links to reliable videos; this may foster a sense of collegiality by promoting group studying. Providing access to reliable videos could overcome potential barriers to watching videos virtually as a group, such as poor Internet connection or inattention. It also allows for repeated practice.^10^

The virtual strategies included in a plan such as this could help to ensure that each student sees a certain minimum number of different examination techniques, abnormal findings, clinical problems, or patients. As mentioned earlier, this could help as a form of standardization and is probably easier to accomplish virtually, rather than providing in-person tutors with a “wish list” of objectives.

The results may be valuable in a broader curriculum context. For example, the expert consensus that certain elements of the neurologic examination are most reliably taught in an in-person setting could allow neurologists to advocate for the importance of in-person teaching even during a pandemic. Conversely, the multiple strategies for teaching the neurologic examination virtually that are presented here could help medical faculties resist the urge to resume in-person teaching too early in a future pandemic.

The results may be valuable for future curriculum design. For example, strategies for teaching the neurologic examination virtually could lead to the development of more effective instructional videos for the neurologic examination. Valuable in-person teaching time could then be used to focus on those elements for which effective strategies do not exist. The use of such blended strategies has been shown to help students learn the neurologic examination.^12,14,25^ Ideally, these strategies could also be used throughout a longitudinal curriculum of undergraduate Neurology education.^26^ One approach is what has been referred to as the “core and cluster” method in which more junior students are taught a basic or essential examination (the “core”) and more senior students are taught how to adapt this to disease or problem-specific clusters.^4,27^ One cluster is a set of questions and/or examination techniques that are specific to a given clinical problem (e.g., eliciting rigidity and bradykinesia in suspected Parkinson disease). Our results emphasize that the ability to adapt the examination is one of the important nuances of learning the examination. Figure provides an overview of how both in-person and virtual teaching of the neurologic examination could be combined in a core and cluster approach.

**Figure:**
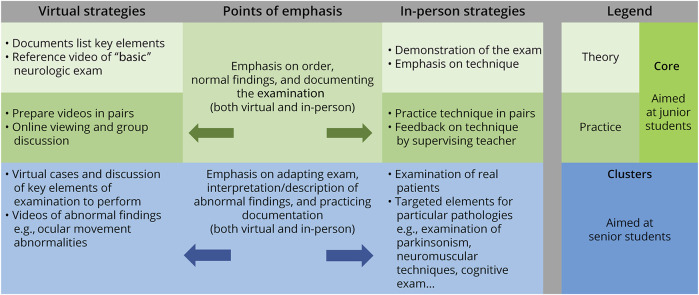
Core and Cluster Approach to Teaching the Neurologic Examination “Core” strategies (in green shades) are intended to help junior students learn a basic or essential examination. They are separated into “theory” (lighter green) and more hands-on “practice” (darker green) strategies. “Cluster” strategies are for more senior students and are specific to different clinical problems. Arrows are used to indicate that the points of emphasis apply to both virtual and in-person strategies.

Finally, there is the potential to apply strategies to global neurologic education. Medical schools in developing countries in which access to specialist educators is limited could benefit from a blended curriculum, in which virtual sessions could be supplemented by less frequent in-person teaching sessions. Virtual platforms have been used to provide education at a national level^12^ and could be applied internationally. However, there may be locations in which a computer and stable Internet access are not routinely available for students.^28^ The cost of more advanced technology such as virtual reality may also be prohibitive.^10^ Cheaper alternatives such as prerecorded teaching sessions might be used. Teaching the neurologic examination may assume even greater importance in such settings because access to advanced imaging and other diagnostic tools may also be limited.

There are limitations to this study. The sufficiency or adequacy of the sample (number of participants) was not predetermined in a specific manner. We also did not use a specific method to assess data saturation. If we had held multiple small focus groups (with different participants), we may have discovered other ideas that were missed here. Our hope was that the diversity of participation in terms of stages of training (3 staff neurologists with extensive experience in medical education, a more junior staff neurologist, a senior neurology resident, and a junior neurology resident with an interest in medical education) allowed us to capture a range of ideas. Opportunities for reflection (after returning to in-person teaching in 2021 and on the survey results) were intended to encourage a broad, nuanced perspective. Participants had ample opportunity to provide their own perspective and respond to others. However, we also acknowledge that these are the perspectives of the authors. Each of us may have had preexisting biases, and these may have shaped the reporting of the findings.

Our study was based on teaching relatively inexperienced second-year medical students. If the focus group participants had also been asked about teaching more senior students or residents, then this may have provided additional insights. In fact, participants did make reference to other teaching experiences. The applicability of our findings may also be limited because we are one group of experts from a single medical school. Our participants may have inherent biases in favor of teaching the neurologic examination in either a virtual or in-person format. A conscious effort was made in the focus group to encourage discussion of advantages and disadvantages of various approaches, rather than simply arguing for the best approach. Recall bias was also a possibility, given the delay between the virtual small group teaching and the focus group. Finally, we do not have actual evidence of the effectiveness of the strategies discussed in this study.

Our original aim was to identify strategies for teaching specific elements of the neurologic examination and develop expert consensus as to what could or could not be taught virtually. However, the ultimate benefit of our results lies in making more explicit what it means to learn the neurologic examination. It is truly a multifaceted process that should emphasize not only technique but also an organized approach to performing and documenting the examination. Virtual education strategies can play a useful role.

## Appendix Authors

**Table:**

Name	Location	Contribution
Sandra Reiter-Campeau, MD	Department of Neurology and Neurosurgery, McGill University, Montreal, Quebec, Canada	Drafting/revision of the article for content, including medical writing for content; major role in the acquisition of data; and analysis or interpretation of data
Stuart Lubarsky, MD	Department of Neurology and Neurosurgery, and Institute for Health Sciences Education, McGill University, Montreal, Quebec, Canada	Drafting/revision of the article for content, including medical writing for content; major role in the acquisition of data; and analysis or interpretation of data
Colin H. Chalk, MD	Department of Neurology and Neurosurgery, and Institute for Health Sciences Education, McGill University, Montreal, Quebec, Canada	Drafting/revision of the article for content, including medical writing for content; major role in the acquisition of data; and analysis or interpretation of data
Asli Buyukkurt, MD	Department of Neurology and Neurosurgery, McGill University, Montreal, Quebec, Canada	Drafting/revision of the article for content, including medical writing for content; major role in the acquisition of data; study concept or design; and analysis or interpretation of data
Myriam Levesque-Roy, MD	Department of Neurology and Neurosurgery, McGill University, Montreal, Quebec, Canada	Drafting/revision of the article for content, including medical writing for content; major role in the acquisition of data
Ana Clouatre	Faculty of Medicine, McGill University, Montreal, Quebec, Canada	Analysis or interpretation of data
Diana Benea, MD	Faculty of Medicine, McGill University, Montreal, Quebec, Canada	Analysis or interpretation of data
Tasnia Rahman, MD	Department of Neurology and Neurosurgery, McGill University, Montreal, Quebec, Canada	Analysis or interpretation of data
Fraser Moore, MD	Department of Neurology and Neurosurgery, and Institute for Health Sciences Education, McGill University, Montreal, Quebec, Canada	Drafting/revision of the article for content, including medical writing for content; major role in the acquisition of data; study concept or design; and analysis or interpretation of data

## Glossary

COVID-19: coronavirus disease 2019
OSCE: objective structured clinical examination
